# Risk factors for paclitaxel-induced peripheral neuropathy in patients with breast cancer

**DOI:** 10.1186/s12885-018-4869-5

**Published:** 2018-10-05

**Authors:** Zohreh Ghoreishi, Seyedali Keshavarz, Mohammad Asghari Jafarabadi, Zahra Fathifar, Karyn A Goodman, Ali Esfahani

**Affiliations:** 1 Nutrition Research Center, Department of Clinical Nutrition, School of Nutrition, Tabriz University of Medical Sciences, Tabriz, Iran; 2 Department of Clinical Nutrition, School of Nutritional Sciences and Dietetics, Tehran University of Medical Sciences, Tehran, Iran; 3 Road Traffic Injury Research Center, Tabriz University of Medical Sciences, Tabriz, Iran; 4 School of Health, Tabriz University of Medical Sciences, Tabriz, Iran; 5 Department of Radiation Oncology, School of Medicine, University of Colorado, Aurora, CO USA; 6 Hematology and Oncology Research Center, Tabriz University of Medical Sciences, Shahid Ghazi Hospital, Tabriz, Iran

**Keywords:** Peripheral nervous system diseases, Paclitaxel, Breast neoplasms, Receptors, progesterone

## Abstract

**Background:**

Paclitaxel induced peripheral neuropathy (PIPN) is a major debilitating side effect of paclitaxel in patients with breast cancer with no fully known mechanisms. The aim of the study was to find out the possible risk factors for PIPN.

**Methods:**

Eligible patients with node positive breast cancer undergoing chemotherapy with paclitaxel were assessed. They belonged to an initial randomized controlled trial in which the effectiveness of omega-3 fatty acids in preventing and reducing severity of PIPN was evaluated (protocol ID: NCT01049295). Reduced total neuropathy score (r-TNS) was used for measuring PIPN. All analyses were performed adjusting for intervention effect. The association between age, BMI, BSA, pathological grade, molecular biomarkers and PIPN was evaluated.

**Results:**

Fifty-seven patients with breast cancer were investigated. Age was significantly associated with risk of PIPN (RR:1.50, *P* value = .024). Body mass index and BSA had significant association with severity of PIPN (B:1.28, *P* = .025; and B: 3.88, *P* = .010 respectively). Also, BSA showed a significant association with the risk of PIPN (RR: 2.28, *P* = .035; B: 3.88, P = .035). Incidence and severity of PIPN were much more pronounced in progesterone receptor positive (PR^+^) patients (RR:1.88, *P* = .015 and B:1.54, *P* = .012). Multivariate analysis showed that age and the status of PR^+^ were independent risk factor for incidence and the status of PR^+^ was the only independent risk factor for severity of PIPN.

**Conclusion:**

Age, BSA and the status of PR^+^, should be considered as the risk factors for PIPN before commencement of chemotherapy with paclitaxel in patients with breast cancer. Older patients, those with greater BSA and PR^+^ patients may need closer follow up and more medical attention due to greater incidence and severity of PIPN.

## Background

Paclitaxel induced peripheral neuropathy (PIPN), is the main dose-limiting and long lasting side effect of paclitaxel, with no fully understood mechanisms. Microtubules aggregation in axons and Schwan cells maybe underlying the sensory axonal peripheral neuropathy due to paclitaxel, while motor and autonomic nervous system are less affected [1–3].

Age, body mass index (BMI), and body surface area (BSA) pathological grade and molecular biomarkers may be related to peripheral neuropathy, there are some adverse changes in the peripheral nervous system observed with aging, and higher doses of neurotoxic medications for those with greater body surface area which may results in more severe neurotoxicity and patients with higher grade of the disease may experience more side effects due to chemotherapy. The aim of this study was to evaluate the possible association between the age, BMI, BSA, pathological grade, and molecular biomarkers of estrogen and progesterone receptors (ERs and PRs), tumor protein p53 and human epidermal growth factor receptor 2 (HER2) with PIPN in order to help oncologists make more accurate medical decisions being tailored to the individual patients based on their characteristics.

## Methods

### Patients

The results of the current study were obtained from a randomized double-blind placebo controlled trial, in which we studied the prophylactic effect of n-3 polyunsaturated fatty acids on incidence and severity of PIPN in female patients with a node positive breast cancer undergoing chemotherapy with 4 courses of 175 mg/m^2^paclitaxel,(protocol ID: NCT01049295) [4]. Other inclusion criteria were having functional kidney and liver, and WHO performance scores of 0–1.

Patients were excluded from the study if they had diabetes mellitus, prior polyneuropathy due to any reasons, had received neurotoxic drugs before chemotherapy with paclitaxel, presence of obvious abnormalities in their nerve conduction studies (NCS) before the onset of treatment, and poor performance status. Paclitaxel (Ebetaxel, Ebewe Pharmaceutical Company, Austria), at a dose of 175 mg/m^2^ based on a 3-h infusion every three weeks for 4 cycles was prescribed for all the patients. The ethical committee of Tehran University of Medical Sciences approved the study (No: 9683) and written informed consent was received from all the patients before the intervention.

### Neurologic assessment

For all the recruited patients, development and severity of PIPN was assessed by the same neurologist. The tool used for measuring PIPN was reduced Total Neuropathy Score (r-TNS) that consists of both clinical and electrophysiological parameters including subjective sensory symptoms, deep tendon reflexes, pin sensibility and sensory action potential amplitude of sural nerve (a-SAP) as well as amplitude of compound muscle action potential (a-CMAP) of peroneal nerve.

Reduced-TNS had 7 items which scored from 0 to 4 depending on their severity; therefore, each study patient got a score from 0 to 28. The grading of PIPN was as follows: grade 0, no PIPN; 1–10, mild; 11–19, moderate; and 20–28, severe PIPN.

Electrophysiological studies were conducted unilaterally (right side) under a standard method [5] using a Nicolet/VIASYS Viking Quest EMG Machine.

### Other measurements

Body mass index and BSA was calculated by Quetelet and Mosteller formulas respectively. Pathological grade was obtained from pathology reports filed in the patients’ medical records, and the status of molecular biomarkers including the status of ER, PR, p53 and HER2 was assessed based on immunocytochemistry method (IHC) by the following kits respectively: FLEX, Monoclonal Mouse Anti-Human Estrogen Receptor α, Clone 1D5, Ready-to-Use, (Dako Autostainer /Autostainer Plus), No cat. IS657 (Dako, Denmark); FLEX, Monoclonal Mouse.

Anti-Human Progesterone Receptor, Clone PgR 636, Ready-to-Use (Dako Autostainer/Autostainer Plus), Catalog No.IS068 (Dako, Denmark); Polyclonal Rabbit, Anti-Human c-erbB-2 Oncoprotein, Catalog No A0485 (Dako, Denmark); FLEX, Monoclonal Mouse Anti-Human p53 Protein, Clone DO-7, Ready-to-Use, (Dako Autostainer/Autostainer Plus), Reference IS616 (Dako, Denmark).

### Statistical analysis

Quantitative and qualitative variables were summarized using mean and standard deviation (SD) and frequency (percent) respectively. Age was categorized into three equal percentiles: ≤ 40, 41–50, and > 50 years old and BMI was divided into two categories based on its median, ≤ 42.95 kg/m2, and > 42.96 kg/m2. The status of steroid receptors, P53, and HER2 was described as positive/negative. Zhang and Yu correction method was used for estimation relative risk (RR) of PIPN incidence in association with selected factors from odds ratio derived from logistic regression analysis [6]. The relationship between these parameters and severity of PIPN was assessed using ordinal regression analysis; the effect size of this model is B, the positive and negative of this coefficient represents direct and inverse relationship respectively. All estimations were performed adjusting for intervention effect. The tests were two-sided, *P* < .05 was considered as significant. SPSS software (SPSS Inc., Chicago, IL) was used for all analysis. The sample size calculation was explained in the original study [4].

## Results

Fifty-seven female patients with node positive breast cancer completed the study. The mean (SD) of age, BMI, and BSA of the patients were 45.76 (10.73) year, 45.07(8.86) kg/m^2^, and 1.73 (.21) m^2^ respectively. Twenty-one patients (37%) were ≤ 40 years old while 19 patients (33%) were between 41 and 50 years, and 17 patients (30%) were over 50 years old. Thirty patients (53%) had BMI ≤ 42.95 kg/m^2^ and 27 patients (48%) had BMI over than 42.96 kg/m^2^. Fifty-eight percent (*n* = 33) of the patients did not develop peripheral neuropathy while 42% (*n* = 24) had some degrees of PIPN, so that the frequency of mild, moderate, and severe PIPN was 24% (*n* = 14), 16% (*n* = 9), and 2% (*n* = 1) respectively (Table 1). The status of pathological grade and molecular biomarkers including: sex hormone receptors, p53, and HER2 was shown in Table 2.

**Table 1:** General characteristics of the patients

Age (year)	45.76 (10.73)*
≤ 40, n (%)	21 (37)
41–50, n (%)	19 (33)
51+, n (%)	17 (30)
Body mass index (kg/m^2^)	45.07(8.86)*
≤ 42.95, n (%)	30 (53)
42.96+, n (%)	27 (47)
Body surface area (m^2^)	1.73 (.21)*
PIPN^**^ n (%)	
None	33 (58%)
Mild	14 (24%)
Moderate	9 (16%)
Severe	1 (2%)
Estrogen receptor positive (%)	52
Progesterone receptor (%)	54
P53 negative (%)	70
HER2 negative (%)	79
Grade (1, 2, 3) (%)	17, 53, 30

*Mean (standard deviation)

**Paclitaxel-induced peripheral neuropathy

**Table 2:** Association between age, BMI, BSA, pathological grade, and molecular biomarkers with PIPN^a^

	PIPN Incidence	PIPN Severity
	RR^b^ (95%CI), *P* value	B (95% CI), *P* value
Age (year)	1.50 (1.07 to 1.87), .024	.61 (−.05 to 1.27), .071
Body mass index (BMI, kg/m^2^)	1.44 (.77 to 1.98), .208	1.28 (.16 to 2.41), .025
Body surface area (BSA, m^2^)	2.28 (1.14–2.38), .035	3.88 (.93 to 6.82), .010
Progesterone receptor (+ vs.-)	1.88 (1.19–2.22), .015	1.54 (.33–2.74), .012

In multivariate analysis, age and progesterone receptor were shown as independent risk factors for incidence of PIPN, RR: 1.57, 95% CI: (1.03 to 1.98), *P* = .037 & RR: 1.85, 95% CI: (1.07 to 2.23), *P* = .034 respectively. Additionally, progesterone receptor was the only independent risk factor for severity of PIPN, B: 1.24, 95% CI: (.01 to 2.47), *P* = .048

^a^Paclitaxel-induced peripheral neuropathy

^b^Relative risk

### Paclitaxel induced peripheral neuropathy

The age was significantly associated with incidence of PIPN adjusting for intervention effect; RR: 1.50, 95% CI = (1.07 to 1.87), *P* = .024, and a trend for significant direct association with PIPN severity, B = .61, 95% CI = −.05 to 1.27, *P* = .071. Also, there was a statistically positive relationship between BMI and PIPN severity, B = 1.28, 95% CI = (.16 to 2.41), *P* = .025. Notably, there was a positive association between BMI and incidence of PIPN without statistical significance, RR: 1.44, 95% CI = (.77 to 1.98), *P* = .208, so that in patients with greater BMI, relative risk of PIPN incidence was 44% higher than those who had lower BMI. Body surface area had statistically significant association with incidence and severity of PIPN, RR: 2.28, 95% CI = (1.14 to 2.38), *P* = .035; and B = 3.88, 95% CI = (.93 to 6.82), *P* = .010 (Table 2). All analysis was adjusted for intervention effect.

In terms of the steroid receptors status, PIPN was significantly more pronounced in progesterone receptor positive (PR^+^) patients, RR: 1.88, 95% CI = (1.19 to 2.22), *P* = .015; and B = 1.54, 95% CI = (.33 to 2.742), *P* = .012. There was no statistically significant association between grade of the disease, the status of P53, and HER2 with PIPN (data not shown).

In multivariate analysis considering age, BMI, BSA, progesterone receptor status and intervention effect, age and progesterone receptor status were independent predictors of the risk for PIPN (RR: 1.57, 95% CI = (1.03 to 1.98), *P* = .037 and RR: 1.85, 95% CI = (1.07 to 2.23), *P* = .034 for older age and PR^+^ status, respectively). Moreover, only PR^+^ status remained the predictor of PIPN severity (B: 1.24, 95% CI: (.01 to 2.47), *P* = .048).

## Discussion

We assessed the association between the age, BMI, BSA, pathological grade, and molecular biomarkers of ERs and PRs, p53 and HER2 and PIPN in patients with node positive breast cancer. In univariate analysis, age, BSA and progesterone receptor status were associated with PIPN while in multivariate analysis, age and PR status were remained as the independent risk factors for the incidence of PIPN and PR^+^ status was the only predictor of PIPN severity.

Our results are in line with many studies that showed advanced age is a significant risk factor for incidence and severity of neurotoxicity induced by chemotherapeutic agents including platinum compounds, bortezomib, thalidomide and paclitaxel in particular, in patients with breast, ovarian, and lung cancer [7–12].

The expression of cyto-skeletal proteins as well as axonal transport decrease in peripheral nerves with aging and advanced age has significant effects on nerve conduction parameters in peripheral nerve system causing clinical symptoms such as reduced sensory distinction and muscle strength. Another matter of concern in older patients is the delay in nerve fibers repair and regeneration after injury that may be due to some changes in neuronal cells including axons and Schwan cells and macrophage reactions as well [13]. However, the average age of our patients was about 46 years, maybe due to the fact that age incidence of breast cancer in Iran is much lower than its global average; therefore, some unknown mechanisms may underline the association of age with PIPN in patients with breast cancer.

Higher BMI was considered as a predisposing factor for oxaliplatin induced polyneuropathy in patients with colorectal cancer [14]. Additionally, in a study performed by Alejandro et al. aiming predicting oxaliplatin induced neuropathy in cancer patients, BSA > 2.0 m^2^ and higher BMI were associated with neurotoxicity in univariate analysis and BSA > 2.0 m^2^ was shown as an independent risk factor [15]. That is in accordance with the results of the current study which suggested a stronger association of BSA with PIPN rather than BMI. However, BMI had a significant relationship with severity of PIPN and it was previously shown as a risk factor for some types of neuropathies such as diabetic neuropathy [16, 17], ulnar neuropathy-like symptoms (BMI ±30 kg/m2) [18] and carpal tunnel syndrome [19]. Although obese patients are at greater risk for diabetes and the latter is a predisposing factor for peripheral neuropathy, diabetes or prediabetes conditions were among the exclusion criteria of this study. It should be mentioned that medication doses of chemotherapy are calculated based on BSA, therefore the larger the BSA, the higher doses of neurotoxic drugs and it may underline the observed effect of BSA and BMI on PIPN (considering the significant correlation between BSA and BMI, *r* = .920 and *P* < .001). Physical activity level of the patients was not measured in this study and evidences showed the important role of exercise in reducing chemotherapy-induced peripheral neuropathy (CIPN) [20–25]. Maybe the patients with greater BMI had a lower level of physical activity, although it needs to be verified.

One of the advantages of this study was the method used for assessing PIPN, rTNS, which did not rely only on physical examination as the under/over estimation may occur with patients and/or physician interpretation. Reduced TNS is composed of physical examination and NCSs scores; it is an easy to use scale for assessing CIPN in clinical and research settings. It is correlated significantly with common scales used for measuring CIPN such as National Cancer Institute-Common Toxicity Criteria (NCI-CTS), and Eastern Cooperative Oncology Group (ECOG), with exclusion of the motor and autonomic symptoms from extended version of TNS, that occur very rarely with paclitaxel [26].

In the current study, PIPN was much more pronounced in PR^+^ breast cancer patients. We know that progesterone has been documented as a neuroprotective hormone with beneficial effects on central and peripheral nervous system including promoting of myelination, myelin repair and improving the spinal cord and brain injuries [27–29]. Moreover, in an experimental study by Roglio et al., The use of progesterone reduced docetaxel-induced peripheral neuropathy in rats and prevented adverse changes in nerve conduction and consequently, it was considered as neuroprotective steroid in peripheral nerves [29]. On the other hand, in another study by Check et al., progesterone receptor antagonists had a palliative role as pain reduction in patients with advanced types of carcinoma [30]. Notably in this study, PR^+^ patients were older (46.33 ± 9.09 vs. 44.88 ± 12.81), and they had considerable higher BMI (46.95 ± 8.93 vs. 42.88 ± 8.74) and BSA (1.76 ± .19 vs. 1.67 ± .22) than PR^−^ patients and the results showed that all these factors were related to PIPN. However, it is a new finding and needs to be confirmed in the future studies.

## Conclusion

This study highlights age, BSA and PR^+^ in particular as potential risk factors for the incidence and severity of PIPN, therefore they should be considered before the commencement of chemotherapy in patients with invasive breast cancer, considering older patients, those with greater BSA, and PR^+^ patients as candidates for less neurotoxic protocols after confirmation of these results. Obviously, finding successful strategies for preventing and relieving CIPN is an urgent need for patients with cancer undergoing treatment protocols known to damage the peripheral nervous system and the oncologists should tailor their medical decisions to the individual patients based on their characteristics.

Since BSA is used to dose chemotherapy, many people with larger BSA, a high fat mass and a low lean body mass, maybe over-dosed with chemotherapeutic agents and therefore may develop more toxicities. Further researches with larger sample size powered to evaluate steroids receptor status, age, BMI, and BSA as risk factors for CIPN is warranted.

## Abbreviations

a-CMAP: Amplitude of compound muscle action potential
a-SAP: Sensory action potential amplitude of sural nerve
BMI: Body mass index
BSA: Body surface area
CIPN: Chemotherapy-induced peripheral neuropathy
ECOG: Eastern Cooperative Oncology Group
ER: Estrogen receptors
HER2: Human epidermal growth factor receptor
IHC: Immunocytochemistry method
NCI-CTS: National Cancer Institute-Common Toxicity Criteria
NCS: Nerve conduction studies
PIPN: Paclitaxel induced peripheral neuropathy
PR: Progesterone receptor
RR: Relative risk
r-TNS: Reduced Total Neuropathy Score
SD: Standard deviation
